# Mixed neuroendocrine carcinoma and hepatocellular carcinoma in the liver

**DOI:** 10.1002/cnr2.1772

**Published:** 2022-12-22

**Authors:** Haruka Tanaka, Hiroyuki Sugo, Naoki Iwanaga, Michio Machida, Ikuo Watanobe, Hironao Okubo, Shiori Hotchi, Kanako Ogura

**Affiliations:** ^1^ Department of General Surgery Juntendo University Nerima Hospital Tokyo Japan; ^2^ Department of Gastroenterology Juntendo University Nerima Hospital Tokyo Japan; ^3^ Department of Diagnostic Pathology Juntendo University Nerima Hospital Tokyo Japan

**Keywords:** FDG‐PET/CT, hepatocellular carcinoma, liver, neuroendocrine carcinoma

## Abstract

**Background:**

Mixed neuroendocrine carcinoma (NEC) and hepatocellular carcinoma (HCC) is extremely rare, thus radiological features have not been fully clarified.

**Case:**

A male patient (age: 70 years) visited our hospital due to a tumor in the liver. Examination using contrast‐enhanced computed tomography (CT) revealed a tumor (diameter: 5.0 cm) in hepatic segment 5, with early enhancement of the peripheral area and slight internal heterogeneous enhancement in the arterial and delayed phases, respectively. F‐18 fluorodeoxyglucose (FDG)‐positron emission tomography (PET)/CT revealed intratumoral heterogeneity, characterized by increased uptake (standardized uptake value, 12.10) in the corresponding low‐density area detected using enhanced CT relative to the surrounding areas of the tumor. On magnetic resonance imaging, diffusion‐weighted imaging also showed high intensity in the corresponding low‐density area detected using CT. Preoperatively, the patient was diagnosed with HCC and underwent anterior sectionectomy. Pathological findings revealed both HCC and NEC components, and the patient was diagnosed with mixed NEC and HCC. Comparison of component distribution with FDG‐PET/CT revealed an increased uptake area was congruent with the NEC component in the tumor.

**Conclusion:**

In this case, the difference in tumor components affected the uptake in FDG‐PET/CT. Such heterogeneous uptake with an enhanced spot may be useful for suspecting the presence of mixed NEC and HCC in patients with atypical HCC.

## 1 INTRODUCTION

Primary hepatic neuroendocrine carcinoma (PHNEC) is particularly rare, and mixed neuroendocrine carcinoma (NEC) and hepatocellular carcinoma (HCC) is rarer (0.46% of primary hepatic malignancies).^1^ Thus far, the imaging features of mixed NEC and HCC have not been studied in detail, complicating efforts to distinguish this tumor type from other tumors in the liver through imaging.

In this article, we discuss a case of mixed NEC and HCC that demonstrated specific heterogeneous uptake in F‐18 fluorodeoxyglucose (FDG)‐positron emission tomography (PET)/computed tomography (CT).

## 2 CASE PRESENTATION

**FIGURE 1:**
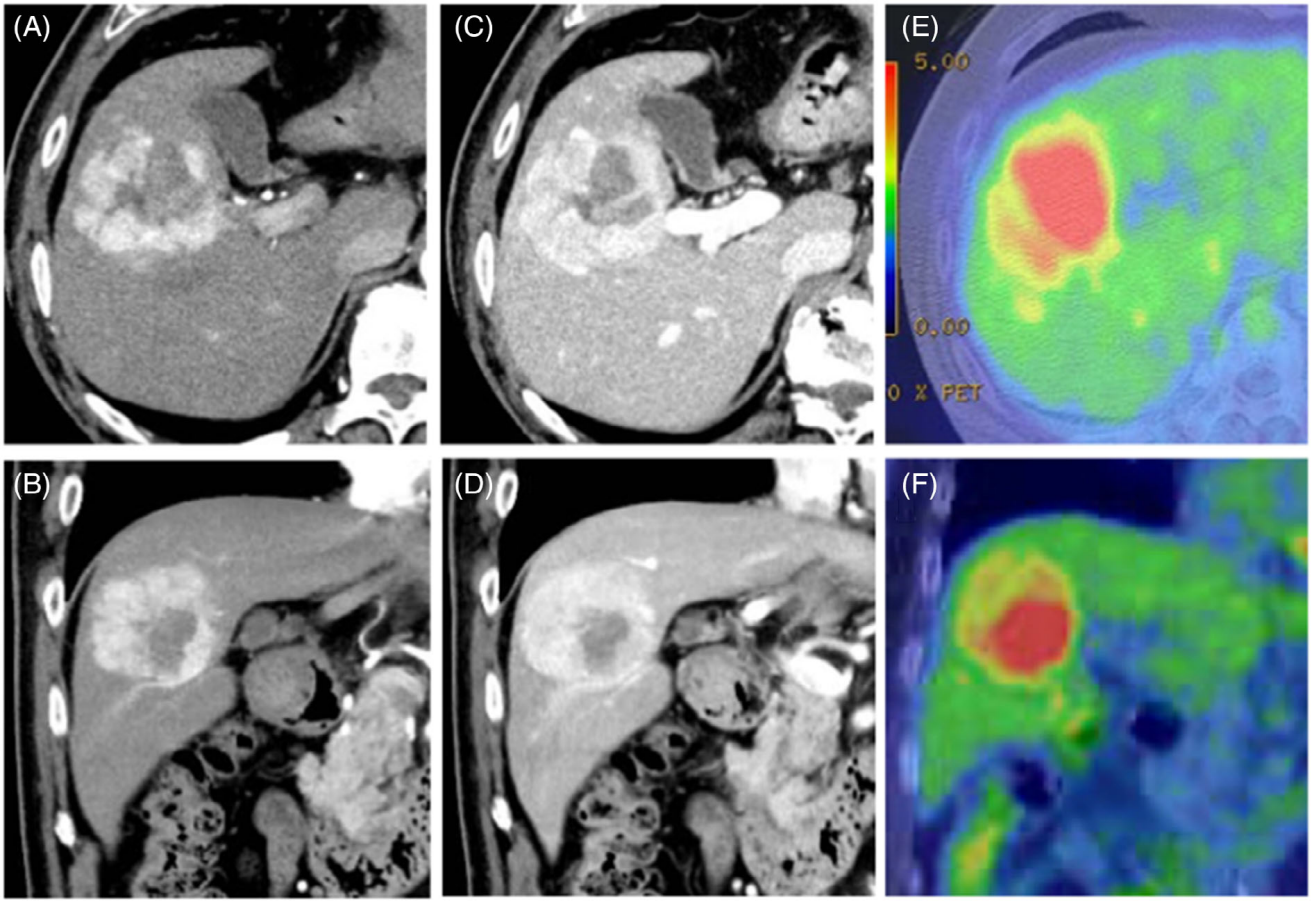
Findings of enhanced abdominal computed tomography (CT) and F‐18 fluorodeoxyglucose (FDG)‐positron emission tomography (PET)/CT: Abdominal CT showing a hepatic mass with early enhancement of the peripheral areas in the arterial phase (A, B) and low‐density structures within the tumor in the delayed phase (C, D). FDG‐PET/CT revealing heterogeneous uptake in the tumor (E, F).

**FIGURE 2:**
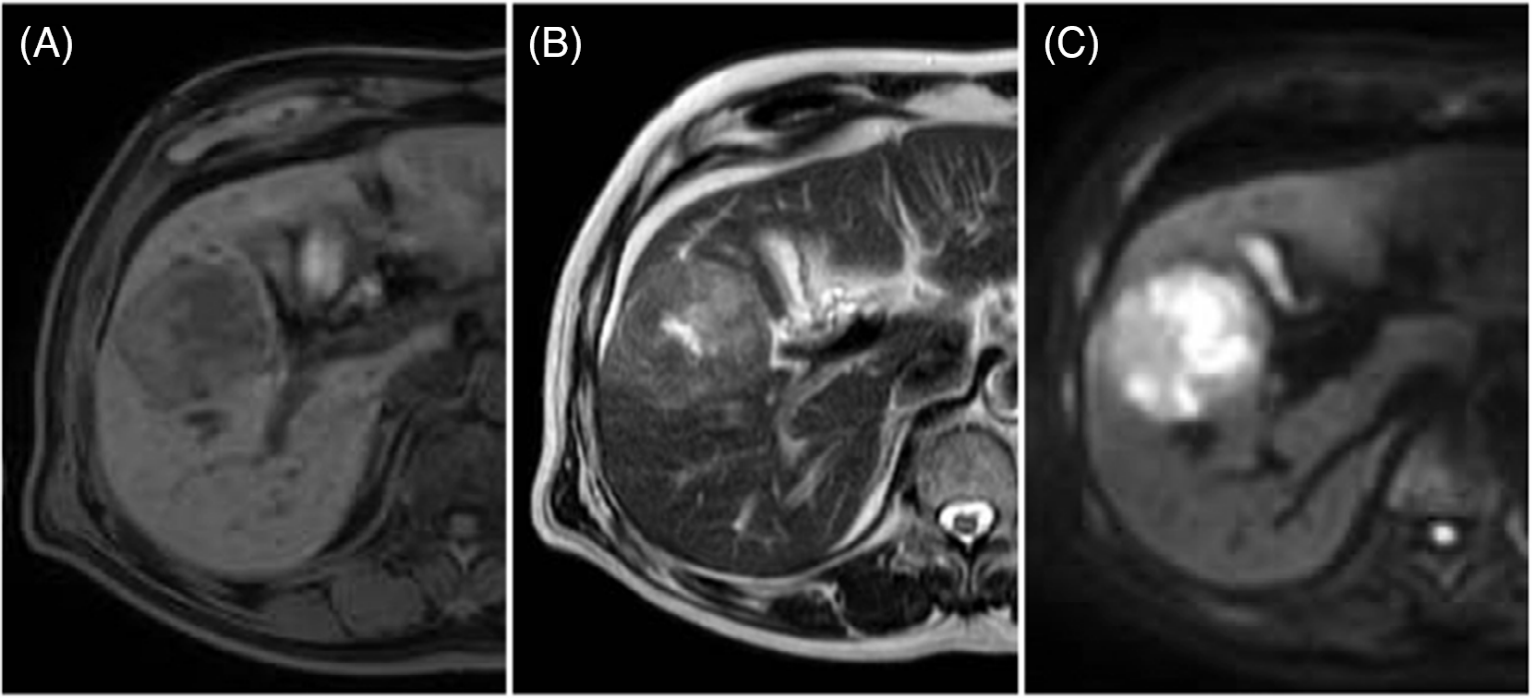
Results of magnetic resonance imaging: Tumor showing low and high signal intensity on T1‐weighted images (A) and T2‐weighted images (B), respectively. Diffusion‐weighted images revealing high signal intensity in the central part of the tumor, corresponding to the low‐density area detected through enhanced computed tomography (C).

**FIGURE 3:**
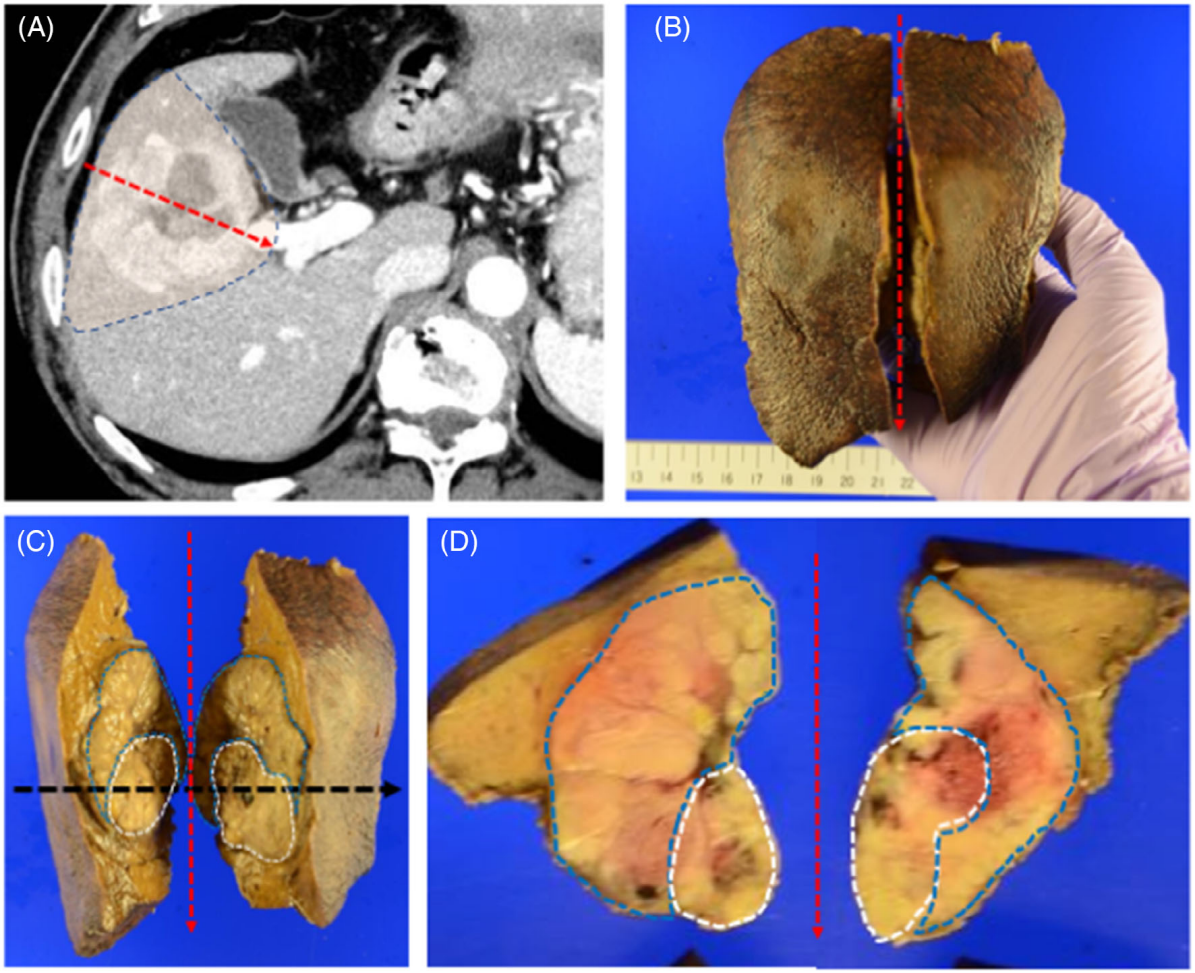
Macroscopic and microscopic features of the resected tumor: CT features of an anterior sector of the liver including the tumor (A). The blue broken line indicates the dividing line used during surgery, and the red broken line shows the cutting line in the resected specimen. Resected specimen (B: above is the head side). The cut surface divided along the red broken line reveals two distinct intermingled components: a whitish area (within the white broken line: NEC area) and a yellowish area (within the blue broken line: HCC area) (C). The horizontal cut surface along the black broken line (C) also shows two distinct components (D).

In July 2021, a male patient (age: 70 years) visited Juntendo University Nerima Hospital due to a tumor in the liver without specific symptoms. He had a normal weight (body mass index 22) and no history of smoking or alcohol consumption. The patient had been receiving outpatient treatment for cerebral hemorrhage at a local clinic, and had a history of diabetes mellitus and hypertension for 3 years. Test results for serum hepatitis B surface antigen and hepatitis C antibody were negative, whereas those for hepatitis B core antibody were positive; the serum levels of alpha‐fetoprotein (AFP) were elevated (1755 ng/ml; normal range: <10 ng/ml). The levels of protein induced by vitamin K antagonist‐II (PIVKA‐II) were also increased (985 mAU/ml; normal range: <40 mAU/ml). Moreover, the levels of other tumor markers (i.e., carcinoembryonic antigen and carbohydrate antigen 19‐9) exhibited slight elevations (6.6 ng/ml; normal range: <5.0 ng/ml and 43.0 U/ml; normal range: <37.0 U/ml, respectively). Examination using contrast‐enhanced CT confirmed the presence of a mass (diameter: 5.0 cm) in the hepatic segment 5, characterized by early enhancement of the peripheral area in the arterial phase (Figure 1A, B) and slight internal heterogeneous enhancement in the delayed phases (Figure 1C, D). FDG‐PET/CT revealed intratumoral heterogeneity, with increased uptake (standardized uptake value [SUV]: 12.10) in a low‐density area detected using enhanced CT relative to the surrounding areas of the tumor (Figure 1E, F). Further investigation through magnetic resonance imaging (MRI) demonstrated low signal intensity and some foci of high signal intensity on T1‐ and T2‐weighted images, respectively. Diffusion‐weighted imaging also showed high intensity in the corresponding low‐density area detected using CT (Figure 2). Whole‐body CT, MRI, and endoscopy did not reveal primary lesions in other sites. Preoperatively, the patient was diagnosed with HCC and underwent anterior sectionectomy. The resected liver specimen weighed 250 g and measured 140 × 110 × 55 mm^3^. It included a well‐defined and heterogeneous friable mass (color: yellow‐white to brown; diameter: 58 mm). In the tumor, two distinct components intermingled with the transitional area and the surrounding tissue was indicative of chronic hepatitis without signs of cirrhosis (Figure 3).

**FIGURE 4:**
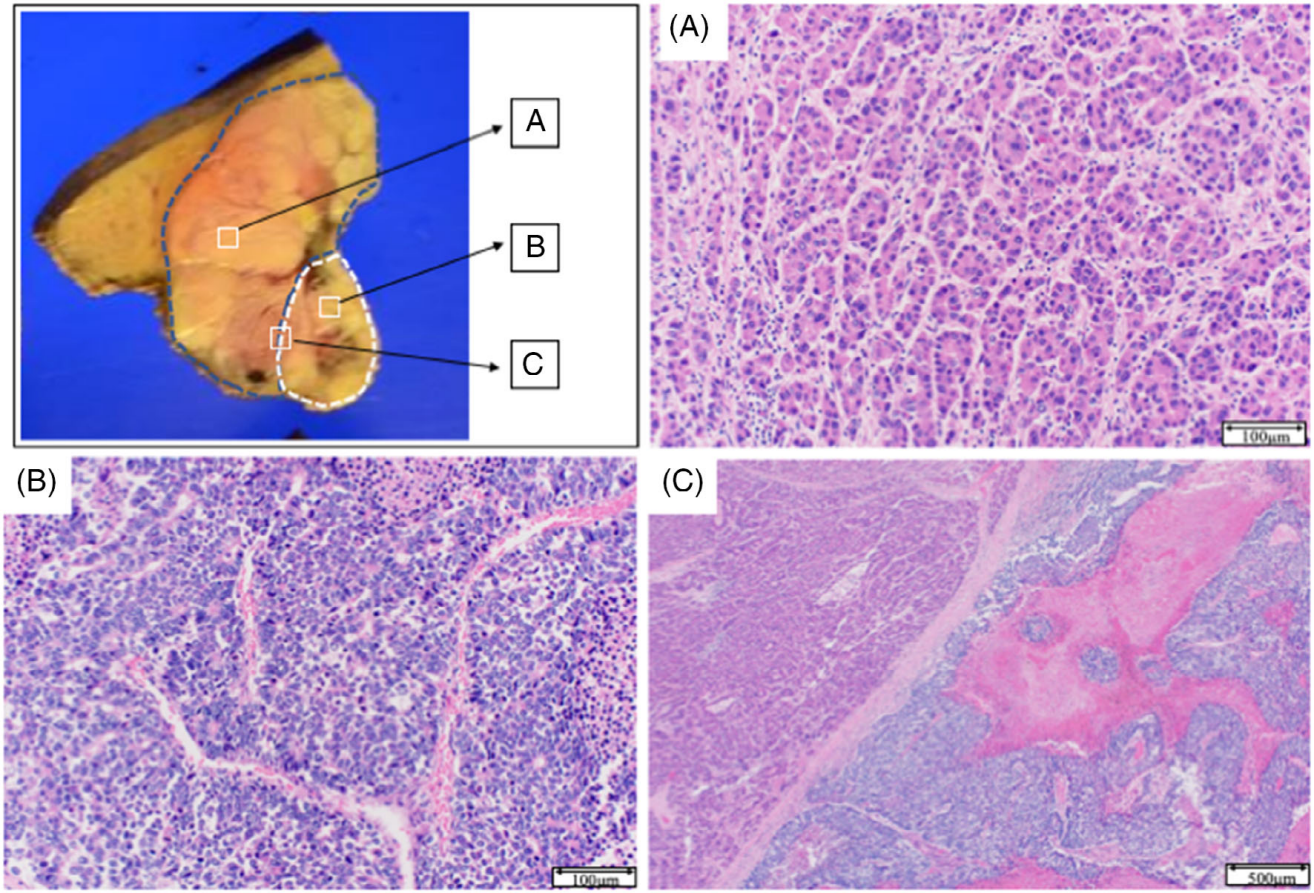
Histological characteristics: The hepatocellular carcinoma‐dominant area is shown inside the blue broken line (A; stain, hematoxylin and eosin stain, magnification, ×200) and the neuroendocrine carcinoma‐dominant area inside the white broken line (B; stain, hematoxylin and eosin stain, magnification, ×400) with transitional area (C; stain, hematoxylin and eosin stain, magnification, ×20).

**FIGURE 5:**
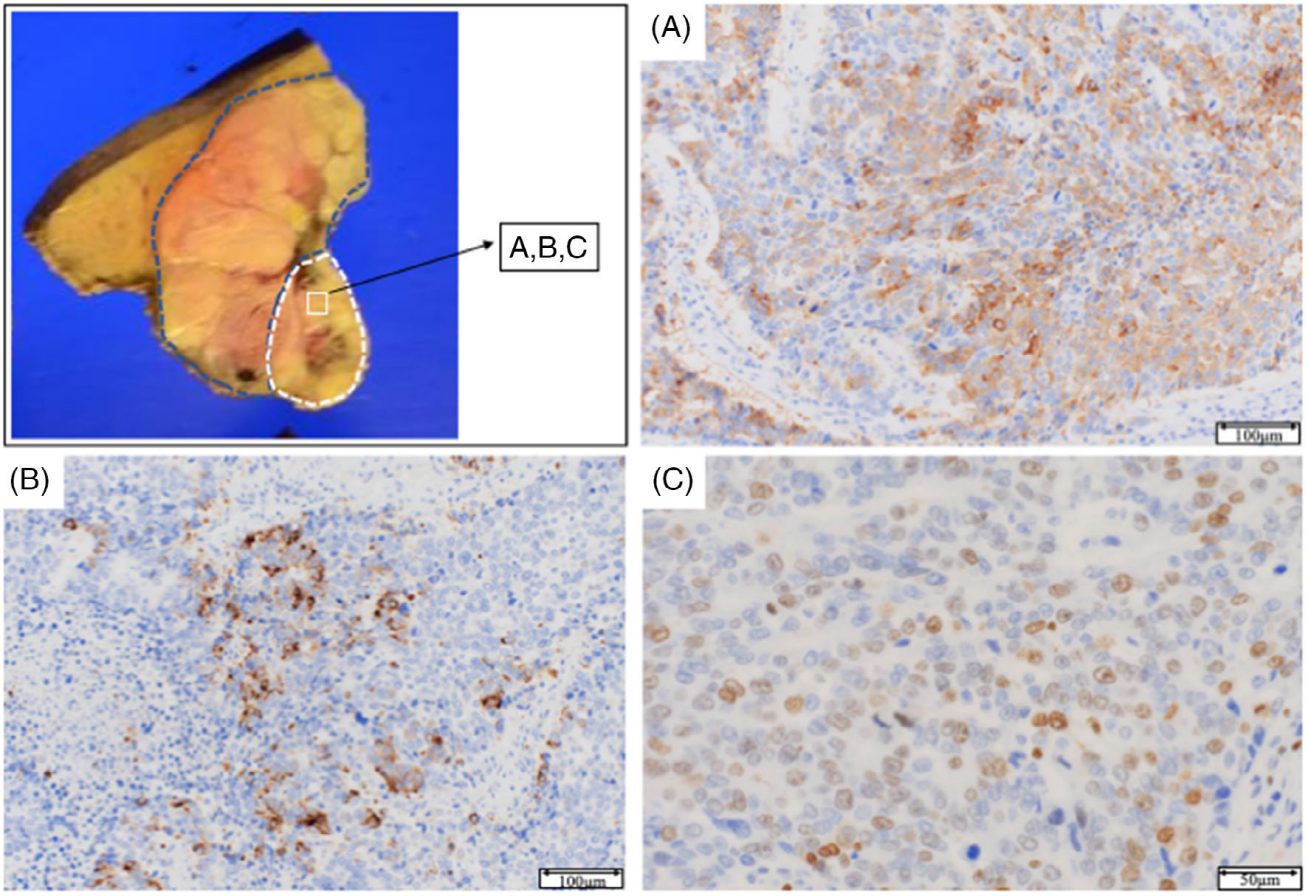
Immunostaining: The neuroendocrine carcinoma component was positive for synaptophysin (A; magnification, ×100) and chromogranin A (B; magnification, ×100), and the Ki‐67 labeling index was approximately 35% (C; magnification, ×400).

**FIGURE 6:**
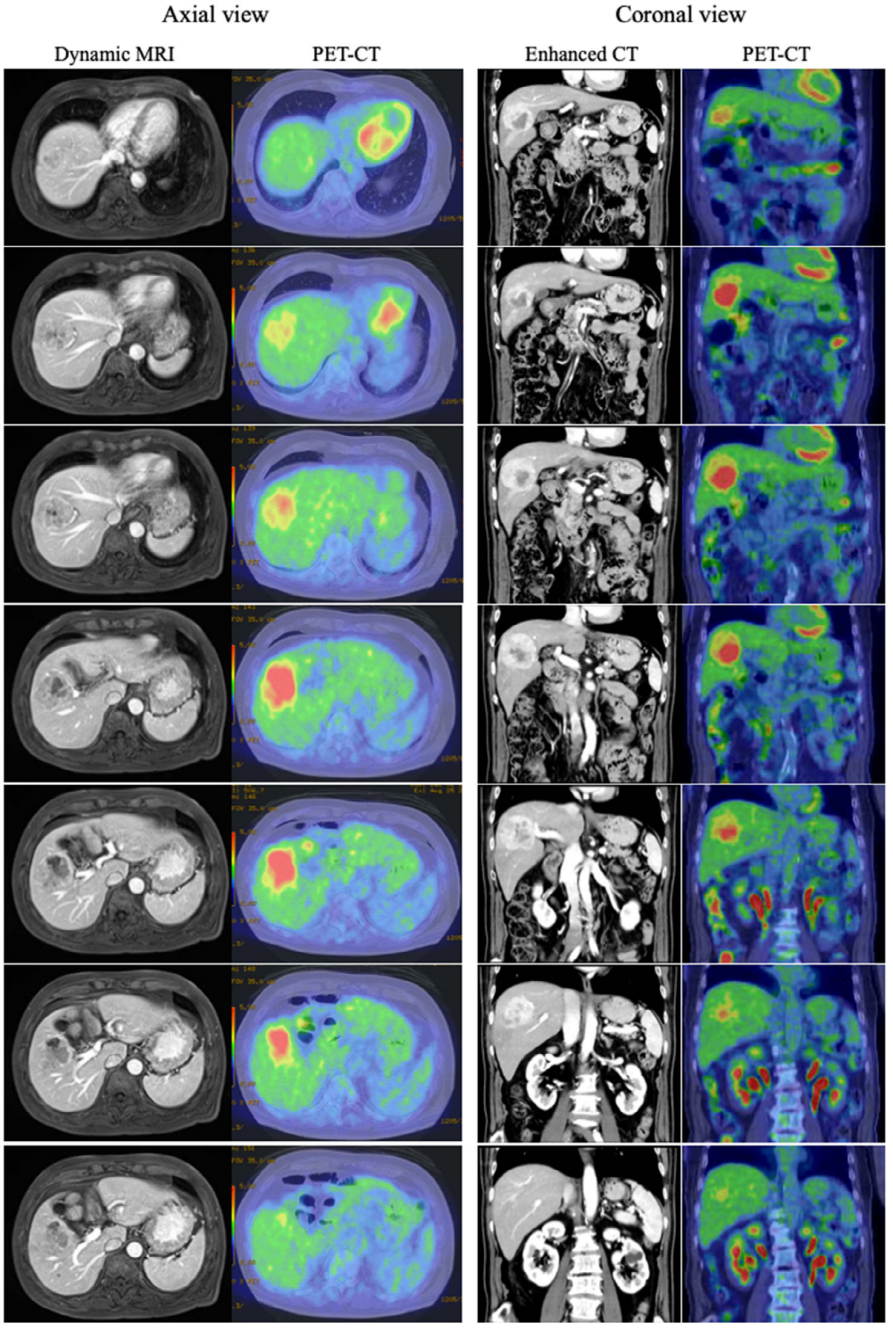
Comparison of findings of PET‐CT with dynamic MRI and enhanced CT. FDG‐PET/CT revealed a strong uptake in the corresponding NEC area in the tumor detected from dynamic MRI and enhanced CT, respectively.

Microscopically, most of the tumor was composed of atypical cells in a trabecular pattern with eosinophilic cytoplasms. These histological features indicated moderately differentiated HCC (Figure 4A). In contrast, gross examination revealed a sheet‐like arrangement of atypical cells in the white‐to‐gray component with a high nucleus‐to‐cytoplasm ratio and chromatin (Figure 4B). Atypical cells with enlarged nuclei and a relatively broad cytoplasm were detected in the region between the two components, potentially constituting a transitional area (Figure 4C). Immunohistochemical analysis of this region showed positivity for synaptophysin and chromogranin A and negativity for hepatocyte paraffin 1 (HepPar‐1). Furthermore, we determined the Ki‐67 labeling index (~35%) (Figure 5). Collectively, these pathological and immunostaining features were indicative of NEC. Based on this histological evidence, the patient was diagnosed with mixed NEC and HCC. On the basis of the Tumor‐Node‐Metastasis (TNM) staging system of the Union for International Cancer Control (UICC), the tumor was considered to be T3N0M0, Stage IIIb. Retrospectively, the findings of PET‐CT are compared with those of dynamic MRI (Axial view) and enhanced CT (Coronal view), respectively (Figure 6). As results, FDG‐PET/CT revealed a congruence in the corresponding NEC area detected from the relationship between imaging, macroscopic features, and pathology. 10 days after surgery, the patient was discharged. At 12 months after surgery, he was in good condition without any CT evidence of tumor recurrence.

## 3 DISCUSSION

Mixed NEC and HCC is very rare condition, thus, only 30 cases have been described as the English literature with detailed information.^2^, ^3^, ^4^ Previously, this tumor involved two types from the differences of pathological findings.^5^ In the first type (combined), the NEC and HCC components are contiguous and intermingled, with a transitional area between them; hence, the separation of these components is challenging. In the second tumor type (collision), the components are histologically distinct. Of note, the combined type is more frequently observed versus the collision type. In the present case, the HCC and NEC components were closely intermingled and their margins were nearly indistinguishable. Thus, the tumor was classified into the combined type.

Distinguishing mixed NEC and HCC from other hepatic tumors is a difficult task. Most previously reported cases mimicked other hepatic tumors and were only identified after surgery. Only two tumors were identified using fine‐needle aspiration and liver biopsy, while one tumor was detected during autopsy.^6^ Preoperatively, most previous cases mimicked HCC because mixed NEC and HCC is commonly accompanied by hepatitis C or B and cirrhosis, along with absence of symptoms. Additionally, Mao et al. demonstrated positivity rates of 77% and 50% for AFP and PIVKA‐II, respectively. In the present case, the patient had hepatitis B and elevated AFP and PIVKA‐II levels, mimicking the preoperative diagnosis of HCC.

Regarding imaging features, Ikeda et al. hypothesized that mixed NEC and HCC showed variation according to the proportions of the components.^4^ In the present case, CT showed only marginal enhancement of the peripheral area and internal heterogeneous enhancement in the arterial and delayed phases, respectively. Diffusion‐weighted imaging in MRI also showed tumor heterogeneity, with high intensity in the corresponding low‐density areas detected using CT. We also performed FDG‐PET/CT preoperatively. FDG‐PET/CT findings for mixed NEC and HCC have not been fully clarified. To date, only one case of mixed NEC and HCC with FDG‐PET/CT findings has been recorded. In that case, FDG‐PET/CT showed a slightly high uptake (SUV, 3.5); however, the tumor was relatively small (diameter: 2.0 cm).^7^ In our case, FDG‐PET/CT showed extremely strong (max SUV, 12.10) heterogeneous uptake. Regarding FDG‐PET/CT findings for PHNEC, Mitamura et al. showed that FDG‐PET/CT sensitivity was dependent on the neuroendocrine tumor (NET) grade, namely 57% and 66% for NET grade 1 and grade 2, respectively.^8^ Previous reports have shown that the sensitivity of this method for the detection of HCC is relatively limited (50%–65%), because of the low sensitivity for well‐ and moderately differentiated HCC.^9^, ^10^ Furthermore, FDG uptake and tumor size are strongly correlated. FDG‐PET/CT exhibits low sensitivity for the detection of HCC with a diameter ≤5 cm; hence, it may not be effective for the diagnosis of such tumors.^11^ Nevertheless, in the present case, FDG‐PET/CT showed an increased uptake, even though the diameter of the tumor was 5 cm. Regarding the degree of FDG accumulation in relation to tumor grade, Tulin et al. reported that the average SUV was 3.51, 3.91, and 9.58 for well‐, moderately, and poorly differentiated HCC, respectively.^12^ In the present case, we assessed the PET uptake values from regions of interest (ROIs) according to tumor components. On the PET images, this showed that the average SUV was 9.9 in the NEC‐dominant area, 3.6 in the HCC‐dominant area, and 2.4 in the non‐tumorous liver. Based on these findings, it was hypothesized that mixed NEC and HCC is associated with higher uptake in FDG‐PET/CT than HCC. Retrospectively, we can consider that our case was atypical for HCC in terms of FDG‐PET/CT findings. Pathological examination showed that the increased uptake area observed in FDG‐PET/CT was comparable to the area of the NEC component in the resected specimen. Consequently, we considered that the heterogeneous intratumoral uptake was due to the difference in the components of mixed NEC and HCC. Ishida et al. reported a case of PHNEC (diameter: 3 cm) in segment 8 that coexisted with HCC (diameter: 1.5 cm) in segment 5.^13^ In that case, FDG‐PET/CT showed high uptake for NEC, but no accumulation for HCC. Their findings are consistent with those of the present case. The difference in uptake degree with FDG‐PET/CT might have been due to the distribution of the tumor components. In this case, we calculated the volume of each of the tumor components using CT volumetry. This revealed that the whole tumor volume was 96.7 cm^3^, and that the volume of the NEC component was 14.5 cm^3^, corresponding to 15% of the whole tumor. Although the NEC component was only 15% in the present case, the increased uptake spot was observed clearly in FDG‐PET/CT.

The clinical significance of tumor components in mixed NEC and HCC has not been fully clarified yet. Several reports have suggested that NEC components are more aggressive than those of HCC, resulting in a high tumor recurrence rate and poor prognosis.^14^, ^15^ Through a review of 28 cases of mixed NEC and HCC, Mao et al. demonstrated median survival and 2‐year survival rates of 17.88 months and 25%, respectively; these rates are worse versus those of HCC.^2^ Nakano et al. suggested that identifying the NEC component is important when considering adequate treatment for patients with mixed NEC and HCC.^15^ In this case, mixed NEC and HCC was associated with heterogeneous intratumoral uptake in FDG‐PET/CT, and the NEC component indicated a strong spot. FDG‐PET/CT is potentially helpful in determining tumor components, thereby possessing prognostic value in patients with mixed NEC and HCC. However, several limitations of this study must be acknowledged. It involved only a single case, and various modalities (e.g., contrast‐enhanced US, EOB‐MRI) were not employed preoperatively, therefore, such definitive interpretation might be difficult to substantiate. On the other hand, the PET‐CT features of mixed NEC and HCC was described in only one patient previously, thus, the radiological features have not been fully clarified. Although further studies with larger numbers of patients are warranted, the present results are compatible with those reported previously, that is, that PET uptake in NEC is higher than that in HCC.^8^, ^13^

## 4 CONCLUSION

In the present case, comparison between the imaging features and pathological findings suggested that the imaging characteristics of mixed NEC and HCC differ according to the volume of the tumor components, and that such differences might affect uptake in FDG‐PET/CT. In the future, heterogeneous uptake with a strong spot in FDG‐PET/CT may be useful for suspecting the presence of mixed NEC and HCC in patients with atypical HCC.

## AUTHOR CONTRIBUTIONS

**Haruka Tanaka:** Conceptualization (lead); investigation (equal); writing – original draft (lead). **Hiroyuki Sugo:** Conceptualization (equal); formal analysis (lead); investigation (supporting); methodology (equal); project administration (lead); supervision (supporting); writing – original draft (lead); writing – review and editing (supporting). **Naoki Iwanaga:** Investigation (equal); resources (lead). **Michio Machida:** Resources (equal). **Ikuo Watanobe:** Writing – original draft (equal); writing – review and editing (equal). **Hironao Okubo:** Investigation (equal); methodology (equal). **Shiori Hotchi:** Writing – review and editing (equal); visualization (equal). **Kanako Ogura:** Investigation (equal); formal analysis (equal); writing – review and editing (equal); visualization (equal).

## CONFLICT OF INTEREST

The authors have stated explicitly that there are no conflicts of interest in connection with this article.

## ETHICS STATEMENT

Written informed consent was obtained from the patient for the publication of case details and use of images. The study was conducted in accordance with the tenets of the Declaration of Helsinki and approved by the ethics committee of Juntendo University Nerima Hospital (Tokyo, Japan; approval number: S22‐05).

## Data Availability

The datasets used and/or analyzed during the current study are available from the corresponding author on reasonable request.
